# Targeting MYC for triple-negative breast cancer treatment

**DOI:** 10.18632/oncoscience.414

**Published:** 2018-06-23

**Authors:** Nancy Klauber-DeMore, Bradley A. Schulte, Gavin Y. Wang

**Affiliations:** Developmental Cancer Therapeutics (DCT) Program, Hollings Cancer Center; Department of Pathology and Laboratory Medicine, Medical University of South Carolina, Charleston, SC 29425, USA

**Keywords:** MYC, cancer stem cells, triple-negative breast cancers (TNBC), triptolide, JQ1

Breast cancer is the most frequently diagnosed form of cancer and the second leading cause of cancer-related death among women in the United States. Triple-negative breast cancer (TNBC) consists of a heterogeneous set of cancers characterized by the lack of hormone receptor expression and human epidermal growth factor receptor 2 (HER2) amplification, and is the most aggressive subtype of breast cancers [1]. Many exciting advances have been made in breast cancer treatment over the past few decades, but effective targeted therapy for TNBC is not available. To make matters worse, drug resistance, metastasis and tumor relapse still frequently occur in TNBC patients after a transient response to initial treatment. This highly unsatisfactory clinic outcome emphasizes an urgent need to develop innovative and more effective therapeutic approaches that can achieve a more durable response in TNBC treatment.

The *MYC* oncogene encodes for a transcription factor, c-Myc (MYC) that is overexpressed in multiple cancer types, including TNBC [2-4]. One of the mechanisms underlying the pro-tumor properties of MYC is likely its function in antagonizing oncogene activation-induced senescence [5]. Many previous studies have demonstrated that various tumors are addicted to MYC and that inactivation of MYC leads to tumor regression in multiple preclinical tumor models [5]. More interestingly, there is evidence that overexpression of MYC contributes to drug resistance in TNBC [3, 4]. Although these elegant studies have suggested strongly that MYC is likely a promising target for TNBC treatment [2-4], therapeutic agents that can directly target MYC have yet to be developed. Our recent studies have demonstrated that the naturally occurring medicinal diterpenoid compound triptolide (C1572) is a new and potent small molecule inhibitor of MYC, that promotes the degradation of the MYC oncoprotein at nanomolar concentrations via a proteasome-dependent mechanism [6]. Strikingly, we found that treatment with C1572 depletes cancer stem cells (CSCs) in TNBC both *in vitro* and *in vivo*, and that the CSC depletion effect of C1572 correlates with a marked inhibition of MYC expression in residual TNBC xenograft tumor tissues. These results suggest that C1572 has considerable potential to be useful in the clinic as a new small molecule inhibitor of MYC to eradicate drug-resistant CSCs for TNBC treatment.

It is worth noting that C1572 is 100-fold more potent than JQ1, a well-characterized and commercially available MYC small molecule inhibitor, in inhibiting MYC in TNBC cells [6]. Moreover, JQ1 functions through an indirect mechanism which interrupts MYC transcription via interacting with one of the BET bromodomain proteins, BRD4 [7]. Thus, the specificity of JQ1 for targeting MYC is a concern. More recent studies in our laboratory have revealed that C1572 can bind to MYC protein, suggesting that C1572 may inhibit MYC via direct targeting. Nevertheless, further in-depth mechanistic studies are warranted to help us better understand how C1572 inhibits MYC in TNBC cells.

A multitude of recent studies have shown that CSCs are resistant to current anticancer therapies, as demonstrated by the fact that they are selectively enriched in residual tumors after chemotherapy [3, 8]. These observations suggest strongly that the inability of current therapies to eliminate CSCs in heterogeneous tumors may account for tumor relapse and treatment failure. Therefore, it has been suggested that new therapeutic agents that can effectively target CSCs may improve the clinical outcome in cancer treatment by eliminating this major root of drug resistance, metastasis and tumor recurrence [8]. The availability and further development of C1572 and/or its derivatives as a new class of MYC small molecule inhibitors would provide a novel and more efficacious therapeutic approach for future clinic management of TNBC and eventually achieve a more durable disease-free patient survival via depleting the drug-resistant and metastatic CSCs.

It has been increasingly recognized that chemotherapy-induced enrichment of CSCs presents a significant but still unmet challenge in cancer treatment [3, 8]. Given the important role of MYC in CSC survival and TNBC drug resistance [3, 4, 6], it is very likely that a combinatorial therapy with C1572 and traditional chemotherapeutic agents such as paclitaxel (Taxol) would be more effective than either agents alone for TNBC treatment, because the depletion of drug-resistant CSCs by C1572 or its derivatives may significantly reduce the risk of metastasis and tumor relapse during the course of treatment. The outcome of the proposed combinatorial therapy in preclinical trials would provide important new information and helpful guidance for the design and planning of future therapeutic regimens for TNBC management.
